# Editorial: Role of p53 in cell metabolism, ferroptosis, and stemness

**DOI:** 10.3389/fgene.2023.1198641

**Published:** 2023-04-14

**Authors:** Jordan Lu, Yanchun Zhang, Jiaxing Yang, Shufang Cui, Jing Zhang, Yanqing Liu

**Affiliations:** ^1^ Herbert Irving Comprehensive Cancer Center, Columbia University, New York, NY, United States; ^2^Department of Genetics and Genomic Sciences, Icahn School of Medicine at Mount Sinai, New York, NY, United States; ^3^ China Pharmaceutical University, Nanjing, China; ^4^ School of Engineering Medicine, Beihang University, Beijing, China

**Keywords:** p53, metabolism, ferroptosis, stemness, cancer, disease treatment

p53 is among the most critical tumor suppressor genes and is the most extensively studied gene in tumor biology within the past 40 years (Dolgin, 2017). Due to its multiple functions in tumor, p53 has been recognized as a promising target to treat cancer (Duffy et al., 2022). In order to leverage the crucial roles of p53 in tumor therapy, it is necessary to illuminate the exact mechanisms for p53 function and how p53 is regulated (Liu et al., 2019). Conventional activities of p53, such as inducing cell cycle arrest, senescence, and apoptosis have been accepted as the major checkpoints in stress responses and tumor suppression for a long period. However, findings in recent years argue against the necessity of these classical activities for p53-mediated tumor suppression (Mello and Attardi, 2018). Accumulating evidence implicates the importance of other mechanisms, including regulating cancer cell metabolism, ferroptosis, and cell stemness to support p53’s anti-cancer role (Liu and Gu, 2022a; Liu and Gu, 2022b). Besides its crucial role in tumor biology, p53 has been demonstrated to function in various other physiological and pathological processes, such as obesity, aging, and immune response. Additionally, the role of mutant p53 and other p53 family members (p63 and p73) are new focuses in research (Mantovani et al., 2019; Osterburg and Dotsch, 2022).

**FIGURE 1:**
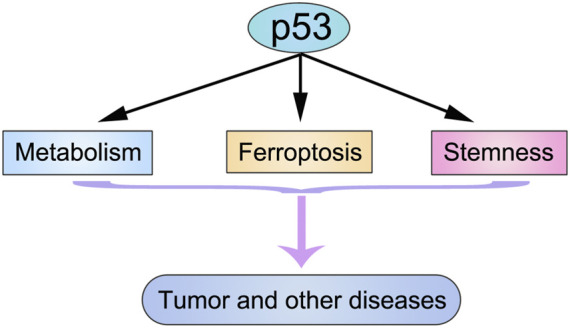
Modulating cell metabolism, ferroptosis, and stemness are three novel functions of p53.

Here we have organized this Research Topic titled “Role of p53 in cell metabolism, ferroptosis, and stemness” and wish to collect manuscripts about the emerging roles of p53 in mediating cell metabolism, ferroptosis, and stemness, which link p53 with diverse diseases, particularly tumors (Figure 1). We have now accepted 11 high quality manuscripts in total. These papers, including research articles and reviews, cover a wide range of Research Topic about p53. We would like to briefly summarize them as follows.

MicroRNA is a type of short non-coding RNA with diverse functions by targeting different genes (Liu et al., 2016; Liu et al., 2017a; Liu et al., 2017b; Liu et al., 2018; Ma et al., 2019). p53 is a master regulator of cell metabolism and is capable of regulating microRNA expression (Hermeking, 2012; Liu and Gu, 2022a). However, the role of the p53-microRNA axis in thermogenesis is largely unknown. Brown adipose tissue (BAT) is the major tissue in mouse to exert non-shivering thermogenesis. Reinisch et al. found that under acute fasting, p53 in BAT was activated to induce the expression of miR-92a-1-5p. They also demonstrated a fructose transporter Slc2a5 to be a repressive target of miR-92a-1-5p. The downregulation of Slc2a5 reduced the import of fructose, glycolysis, and finally thermogenesis. This study revealed a novel p53/miR-92a-1-5p/Slc2a5 axis in fasting-regulated thermogenesis.

Lei et al. in their comprehensive review focused on the role of p53 in the brain. They firstly introduced a basic background of p53 and its regulation. They then discussed how p53 regulates various diseases in the brain, including glioma, cerebral stroke, and neurodegenerative diseases.

Ferroptosis is a newly discovered cell death type, which is caused by the iron dependent peroxidation of cell membranes (Stockwell, 2022). p53 has been proved to be a critical regulator of ferroptosis (Liu and Gu, 2022b). Zhou et al. wrote a short review about ferroptosis and lymphoma, highlighting that targeting the p53-mediated ferroptotic pathway may be a promising method to treat lymphoma. pan and Wang contributed a review about p53 and ferroptosis in osteosarcoma. In this review, they classified ferroptosis into typical and atypical types. In both types of ferroptosis, p53 has a vital role. Ferroptosis also contributes to diseases other than cancer, such as in the regulation of diabetes. In the review by Du et al., authors depicted the major mechanism of ferroptosis and its effect in diabetes. Notably, they pointed out the crucial role of p53-regulated ferroptosis in the pathology of diabetes. In an interesting study from Hu et al., authors investigated the function of a bioactive peptide G1dP3 in MH7A cells. They showed that G1dP3 significantly suppressed the viability of MH7A cells by inducing ferroptosis. Mechanistically, they demonstrated that G1dP3 could activate p53 expression, resulting in the suppression of the SLC7A11/GPX4 axis.

p53 functions as a checkpoint molecule to suppress tumorigenesis. To overcome this, p53 is often mutated or deleted in tumor cells. Mutant p53 may have different functions compared to wild type p53 (Mantovani et al., 2019). Liu et al. presented an informative review about how p53 mutation or deletion remolds the tumor immune microenvironment. There are at least three ways that mutant p53 or loss of p53 contributes to an immunosuppressive status: 1) induce immunosuppressive cytokines and downregulate proinflammatory factors; 2) augment the expression of immunosuppressive ligands; and 3) facilitate immunosuppressive cells differentiation. Correspondingly, targeting mutant p53 would be a reasonable choice to restore the tumor immune microenvironment. Corazzari and Collavin wrote an attractive review to summarize the ferroptosis-regulatory activity of both wild type and mutant p53. They proposed that the regulated ferroptosis could be a stress response to diverse stimuli in and out of the tumor cells. K120 is a critical site for p53 function. Monti et al. analyzed the transactivation activity of the p53 K120R mutant. The K120R mutation abrogates the acetylation at this site, preventing p53 from promoting apoptosis. However, this mutant could still retain some activity to modulate cellular metabolism. Mutant p53 also influences the efficacy of tumor treatment. Li et al. reported a pulmonary sarcomatoid carcinoma case with p53 T125K mutation. Fortunately, treatment of Tislelizumab in combination with Anlotinib enabled this patient to go into complete remission. We hope that this therapeutic regimen may benefit other similar patients.

Other members of the p53 family, such as p63 and p73, also have fundamental roles in tumor (Osterburg and Dotsch, 2022). Signal transducer and activator of transcription 3 (STAT3) is a vital transcription factor in stem cells. The review by Wei et al. discussed the crosstalk between p63 and STAT3 in the regulation of cancer stemness.

To summarize, this Research Topic of papers present novel information about some unconventional functions of p53 (and p63). Elucidating the mechanisms of p53 that underlie its versatile roles will not only benefit the basic research about p53 and tumor biology, but also open promising doors for developing therapeutics targeting p53 and related pathways for the treatment of cancer and other diseases. We hope this Research Topic can bring new knowledge and ideas to those who are interested in the function of p53.
